# The natural history of pregnancies with prenatal diagnosis of Trisomy 18 or Trisomy 13: Retrospective cases of a 23-year experience in a Brazilian public hospital

**DOI:** 10.1590/1678-4685-GMB-2018-0099

**Published:** 2019-06-03

**Authors:** Julio Alejandro Peña Duque, Charles Francisco Ferreira, Suzana de Azevedo Zachia, Maria Teresa Vieira Sanseverino, Rejane Gus, José Antônio de Azevedo Magalhães

**Affiliations:** 1 Hospital de Clínicas de Porto Alegre, Porto Alegre, RS, Brazil; 2 Universidade Federal do Rio Grande do Sul, Porto Alegre, RS, Brazil; 3 Medical Genetics Service, Hospital de Clínicas de Porto Alegre, Porto Alegre, RS, Brazil; 4 School of Medicine, Pontifícia Universidade Católica do Rio Grande do Sul (PUCRS), Porto Alegre, RS, Brazil; 5 Department of Gynecology, Universidade Federal do Rio Grande do Sul, Porto Alegre, RS, Brazil

**Keywords:** Natural history of trisomy, trisomy 13, trisomy 18, prenatal diagnosis, genetic counseling

## Abstract

Trisomy 18 (T18) and trisomy 13 (T13) are polymalformative syndromes associated with a high rate of spontaneous abortions, intrauterine death, and short postnatal life. This study describes the overall outcome in a country where the therapeutic interruption of pregnancy is not available. The medical records of women with prenatal diagnosis of full trisomy of T13 or T18 between October 1994 and October 2017 were analyzed in order to describe their natural outcomes. Thirteen cases of T13 and 29 cases of T18 were included. The miscarriage rate was 9% for T18 and no cases for T13. Intrauterine fetal death occurred in 46% and 52% of cases for T13 and T18, respectively. The rate of live births for T13 was 54%, and the median survival was one day (95% CI -33.55 - 90.40) and 71% died in the first 24 hours of life. The rate of live births for T18 was 37% and the median survival was two days (95% CI -1.89 - 13.17); 90% of the affected babies died within first week of life. For the affected babies reaching the first year of life and for those who lived longer, multiple invasive and expensive procedures were required, without success in prolonging life beyond 180 days. This large series provides information for professionals and women regarding the natural histories of T13 and T18. Results of this study are consistent with those referenced in the literature, emphasizing the need of structured protocols and guidelines aiming early T13 and T18 diagnosis, prenatal care, gestation/parents follow-up, and counseling processes. For those couples with earlier diagnosis, a better follow-up and counseling during the prenatal care lead to the option for a support or palliative management of the newborn. Finally, when the counseling process is appropriate, it becomes easier to take decisions respecting the parent’s autonomy and to look for better outcomes for both, the mother and the fetus.

## Introduction

During the last decade, new screening methods and protocols for prenatal diagnosis of genetic disorders in the first and second trimesters of pregnancy have been established for the general and the at-risk-profile population. Since then, the aneuploidy detection has increased, mainly for trisomies 21, 18 and 13 (Chitayat *et al.*, 2011). Trisomy 18 (T18) is the second most common autosomal aneuploidy in newborns after trisomy 21 (Hook *et al.*, 1983), with a prevalence of 1/3,000 births and trisomy 13 (T13) is the third most common cause of autosomal aneuploidy (Hook *et al.*, 1983), with a prevalence of 1/5,000 births. In Brazil all women can have access to prenatal care, but there is no national policy for universal screening for aneuploidies, which may lead to late prenatal care and late diagnosis.

These severe and potentially lethal polymalformative syndromes are associated with a high rate of spontaneous abortion, intrauterine death, and short postnatal life with early neonatal death (Nicolaides, 2003), due to the presence of multiple anatomical abnormalities, including cardiovascular, neurological, renal, gastrointestinal, and skeletal malformations (Edwards *et al.*, 1960; Patau *et al.*, 1960; Springett *et al.*, 2015). Currently, the reduced prevalence of these disorders in developed countries and the heterogeneity of the studies on diverse populations, leads to a lack of information about the follow-up of T13 and T18 pregnancies, their natural histories, and the overall outcomes after prenatal diagnosis. Another important point is the difference in legislation of termination of pregnancy for fetal conditions in countries where the studies were performed (So *et al.*, 2017). In most of the countries, when a severe fetal malformation or chromosomal disorder is identified, the couple has the opportunity to decide for a medically induced abortion or therapeutic anticipation of delivery. The rates of termination of pregnancy may be above 78% for T13 and T18 (Lakovschek *et al.*, 2011). The natural history of these trisomies is characterized by a high risk of spontaneous fetal loss, with pregnancy loss rates ranging from 49–66% for pregnancies with T13 and between 72–87% for T18 (Morris and Savva, 2008; Lakovschek *et al.*, 2011; Houlihan and O’Donoghue, 2013). Less than 10% of the live births of T13 and T18 have a 1-year overall survival (Lin *et al.*, 2006; Vendola *et al.*, 2010; Houlihan and O’Donoghue, 2013). However, few studies describe isolated cases with greater survival, after performing a large number of interventions (e.g., orthopedic, neurological, cardiac) with poor outcomes and a very precarious quality of life (Redheendran *et al.*, 1981, Petek *et al..,* 2003, Vendola *et al.*, 2010; Bruns and Campbell, 2014). The median survival rate for affected infants was 3 and 15 days for both trisomies (Brewer *et al.*, 2002; Sibiude *et al.*, 2011; Houlihan and O’Donoghue, 2013).

Brazilian legislation criminalizes abortion, and only contemplates interruption of pregnancy in cases of risk of maternal death, fetuses diagnosed with anencephaly, and for cases of rape; in some specific cases a voluntary termination of pregnancy may be asked after judicial authorization. For most of cases with several and potentially lethal malformations, a routine follow-up of pregnancies is performed and the outcome of the natural history is expected.

This study aims to describe the natural history of T13 and T18 after prenatal diagnosis, in a country where the termination of pregnancy for these cases is not legally available, describing the characteristics of the fetuses, the course and final outcome of the gestation. This information should improve the processes of genetic counseling for pre- and postnatal follow-up. Knowledge of the possible outcomes and complications in these conditions may help parents and multidisciplinary teams to make decisions related to the future management of these pregnancies.

## Subjects and Methods

### Subjects

This was a retrospective cohort study, approved by the ethics committee of the Hospital de Clínicas de Porto Alegre in March 2016. The sample size calculation was performed in the WinPEPI version 11.63, based on the study by Lakovoschek *et al.* (2011). Considering the proportion of prenatal diagnosis of T13 (assumed proportion: 0.25) with a sample power of 80% and an acceptable difference of 10%, the final sample size, 31 individuals would be required. Considering the proportion of prenatal diagnosis of T18 (assumed proportion: 0.11) with a sampling power of 80% and an acceptable difference of 10%, the final sample size required would be 17 individuals.

All cases with result of full T13 and full T18 were identified from the personal database of the researchers and the records of karyotype results performed by amniocentesis. The medical records of participants with prenatal diagnosis of T13 and T18 by amniocentesis were analyzed from October 1994 to October 2017, and we included those with complete information about the final outcome of pregnancy. When the medical records were incomplete, the participants were contacted by telephone, using a previously structured script in order to obtain the missing data.

### Data collection and statistical analysis

**Figure 1:**
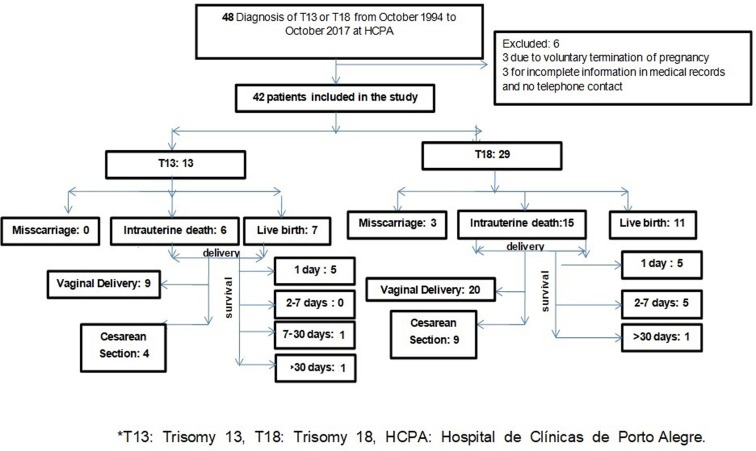
Flowchart of patients diagnosed with trisomy 13 or trisomy 18.

Data collected included demographic information (e.g., age, parity, comorbidities); gestation considerations (e.g., reason for referral to fetal medicine, complications); gestational age at diagnosis of trisomy, type of outcome (e.g., miscarriage, intrauterine death, or live birth); maternal complications, maternal inpatient time, and ultrasound data (e,g., fetal sex detected malformations); gestational age at delivery, delivery route, and overall survival in days for the live births (Figure 1).

The data was collected using a semi structured questionnaire and then imported into SPSS® Statistics Version 18.0 (SPSS Inc. Released 2009. PASW Statistics for Windows, Version 18.0. Chicago: SPSS Inc.). The normality of each variable was evaluated through the Shapiro-Wilk Test. Descriptive statistics were used to present the data. Continuous variables were summarized as medians and 95% confidence intervals (CI) and categorical variables summarized as absolute (n) and relative (n%) frequencies. The Chi-Square test with adjusted residual analysis was performed. Statistical significance was set at 5% for all analyses.

## Results

**Figure 2:**
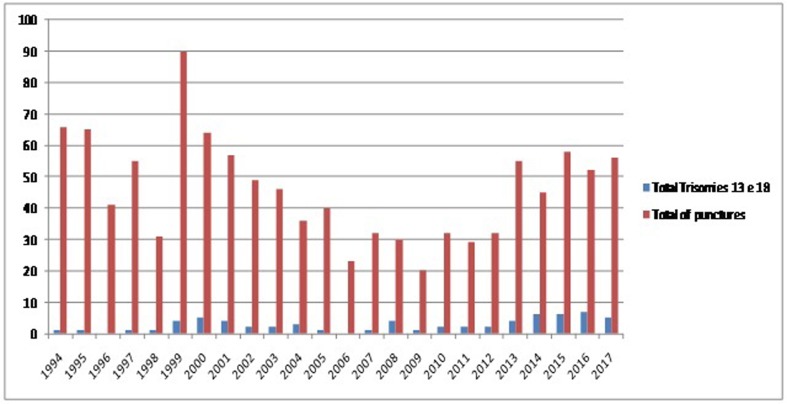
Number of punctures vs. diagnoses of trisomy 13 or trisomy 18 during 23 years.

The local prevalence of T13 and T18 in the Hospital de Clinicas de Porto Alegre, over the last 23 years was 0.15/1,000 births and 0.34/1,000 births, respectively. We had 85,000 births and we performed 1,104 punctures for fetal karyotype. Sixty-five of them (5.8%) had a diagnosis of T13 or T18. Of these 65 exams, there were 20 cases (31%) of full T13 and 45 cases (69%) of full T18 (Figure 2).

### Characteristics of pregnant women

Forty-eight cases had a diagnosis of trisomy during the time of the study. Six of them were excluded. Three had incomplete information and the researchers could not establish contact with them; and the other three performed a voluntary termination of pregnancy after judicial authorization. Forty-two participants were included in the study, 13 (31%) were diagnosed with T13 and 29 (69%) with T18. As shown in Table 1, 47.6% of the women were between 19 and 34 years old, while 45.2% were older than 35 years at the time of trisomic gestation. There was no statistical difference between T13 and T18 (χ^2^, *p*=0.142), but the proportion of women above 35 years was higher for T18 (n=16 of 29, 55.2%) when compared to T13 (n=3 of 11, 23.1%). The median (95% CI) age for T13 was 30 (26.15–35.39) years and 36 (30.85–36.94) years for T18.

**Table 1:** Sample characterization of trisomy 13 and 18 pregnancies.

Variables – *n*(*n*%)	Total *N*=42	T 13 *n*=13	T18 *n*=29	^*^ *p-*value
Maternal age				
≤18 years	3(7.1)	1(7.7)	2(6.9)	0.142
19-34 years	20(47.6)	9(69.2)	11(37.9)	
≥35 years	19(45.2)	3(23.1)	16(55.2)	
Parity				
First pregnancy	14(33.3)	4(30.8)	10(34.5)	1.000
Multiparous	28(66.7)	9(69.2)	19(65.5)	
Gestational age at diagnosis				
1° trimester	1(2.4)	0(0.0)	1(3.4)	0.728
2° trimester	24(57.1)	7(53.8)	17(58.6)	
3° trimester	17(40.5)	6(46.2)	11(37.9)	
Comorbidities				
Yes	8(19.0)	4(30.8)	4(13.8)	0.384
No	34(81.0)	9(69.2)	25(86.2)	
Fetus Sex				
Male	21(50.0)	7(53.8)	14(48.3)	1.000
Female	21(50.0)	6(46.2)	15(51.7)	
Gestational age at delivery				
<22+6 weeks	3(7.1)	0(0.0)	3(10.3)	0.450
23 – 31+6 weeks	10(23.8)	2(15.4)	8(27.6)	
32 – 36+6 weeks	23(54.8)	9(69.2)	14(48.3)	
>37 weeks	6(14.3)	2(15.4)	4(13.8)	
Maternal Complications				
Yes	9(21.4)	4(30.8)	5(17.2)	0.561
No	33(78.6)	9(69.2)	24(82.8)	
Type of Maternal Complications				
None	33(78.6)	9(69.2)	24(82.8)	0.054
Gestational Hypertensive Disorders^**^	5(11.9)	4(30.8)	1(3.4)	
Gestational Diabetes	3(7.1)	0(0.0)	3(10.3)	
Others^***^	1(2.4)	0(0.0)	1(3.4)	
Maternal hospital stay beyond 3 days				
Yes	6(14.3)	2(15.4)	4(13.8)	0.892
No	36(85.7)	11(84.6)	25(86.2)	

Legend: n: absolute frequency; n%: relative frequency; *p*: statistical significance; ^**^gestational hypertensive disorders involve gestational hypertension, pre-eclampsia, and pre-eclampsia superimposed on chronic hypertension; ^**^other complications involve depression, hypothyroidism, and asthma; T13: trisomy 13; T18: trisomy 18; ^*^Chi-Square test with adjusted residual analysis. Statistical significance was set as *p*≤0.05 for all analyses.

Fourteen subjects (33.3%) were primiparous, and there was no statistical difference regarding parity between T13 and T18 (χ^2^ test, *p*=1.000). None of the pregnant women had a history of having a previously conceived pregnancy with T13 or T18 diagnosis. Nineteen percent of the participants had some comorbidity at the time of gestation, but without statistical difference between the two trisomies (χ^2^ test, *p*=0.384). The prevalence of diabetes mellitus was low in our series (Table 1).

### Fetal characteristics and diagnosis of trisomy

Thirteen cases of T13 and 29 cases of T18 were identified, all of them having a prenatal diagnosis with full trisomy by karyotype. No cases of trisomy were identified in twin gestations. The fetal sex distribution did not differ between the two trisomy groups: 50% male and 50% female for both (χ^2^ test, *p*=1.000). Even though the literature describes a ratio of 3:1 female to male fetuses for T18, this proportion was not evidenced in our series.

The gestational age at diagnosis was considered based on the gestational age when amniocentesis was performed. Three categories were defined: the first trimester until 13 weeks + 6 days. Second trimester was defined from 14 to 28 weeks and third trimester from 28 weeks + 1 day. One case (2.4%) was diagnosed in the first trimester, corresponding to the T18 group. Twenty-four cases (57.1%) were diagnosed in the second trimester and the other 17 cases (40.5%) in the third trimester. There was no difference between T13 and T18 gestational age at diagnosis (χ^2^ test, *p*=0.728).

The most common reason for the referral of those women to a specialized follow-up of high risk-profile pregnancy was the finding of fetal malformations by ultrasound, corresponding to 92.9% (39/42) of the cases. The presence of altered nuchal translucency (a nuchal translucency higher than percentile 95) and the risk-profile of malformations were also considered for referral as shown in Table 2.

**Table 2:** Referral reasons for specialized follow-up of high risk-profile pregnancies.

Variables – *n* (*n*%)	Total *N*=42	T13 *n*=13	T18 *n*=29	^*^ *p-*value
Abnormal NT ^**^				
Yes	9(21.4)	1(7.7)	8(27.6)	0.296
No	33(78.6)	12(92.3)	21(72.4)	
Malformations in routine ultrasound				
Yes	39(92.9)	12(92.3)	27(93.1)	1.000
No	3(7.1)	1(7.7)	2(6.9)	
High maternal risk-profile^***^				
Yes	14(33.3)	5(38.5)	9(31.0)	0.906
No	28(66.7)	8(61.5)	20(69.0)	

**Legend:** n: absolute frequency; n%: relative frequency; *p*: statistical significance; ^**^nucal translucency is abnormal when higher than 95 percentile for gestational age; ^***^ high maternal risk-profile is given by age, personal history of the fetus with malformations, and/or personal history of genetic disorders. T13: trisomy 13; T18: trisomy 18; NT: nucal translucency. ^*^Chi-square test with adjusted residual analysis. Statistical significance set as *p*≤0.05 for all analyses.

### Congenital defects

The structural abnormalities detected by ultrasonography or described by the pathologist during autopsy of the fetus with T13 and T18 diagnosis are displayed in Table 3. In total, 30 (71.4%) participants had a cardiac defect (e.g., interatrial communication, interventricular communication, and/or atrioventricular septal defect) detected by ultrasound (T13=53.5%, T18=79.3%). Gastrointestinal malformations were identified in 12 (28.6%) subjects (T13=23.1%, T18=31.9%). Genitourinary malformations were identified in 20 (47.6%) cases (T13=61.5%, T18=41.4%). Malformations of the central nervous system were identified in 27 (64%) participants (T13=84.6%, T18=55.2%). Abnormalities of limbs were present in 46.2% of cases of T13 and 65.5% of T18.

**Table 3:** Structural abnormalities for trisomy 13 and trisomy 18.

Variables – *n*(*n*%)	Total *N*=42	T13 *n*=13	T18 *n*=29	^*^ *p-*value
Cardiac				
Yes	30(71.4)	7(53.8)	23(79.3)	0.187
No	12(28.6)	6(46.2)	6(20.7)	
IAC				
Yes	13(31.0)	4(30.8)	9(31.0)	1.000
No	29(69.0)	9(69.2)	20(69.0)	
IVC				
Yes	20(47.6)	5(38.5)	15(51.7)	0.514
No	20(52.4)	8(61.5)	14(48.3)	
AVSD				
Yes	10(23.8)	2(15.4)	8(27.6)	0.391
No	32(76.2)	11(84.6)	21(72.4)	
Gastrointestinal				
Yes	12(28.6)	3(23.1)	9(31.0)	0.874
No	30(71.4)	10(76.9)	20(69.0)	
Omphalocele				
Yes	11(26.2)	3(23.1)	8(27.6)	1.000
No	31(73.8)	10(76.9)	21(72.4)	
Gastroschisis				
Yes	0(0.0)	0(0.0)	0(0.0)	1.000
No	42(100.0)	13(100.0)	29(100.0)	
Duodenal atresia				
Yes	0(0.0)	0(0.0)	0(0.0)	1.000
No	42(100.0)	13(100.0)	29(100.0)	
Esophageal Atresia				
Yes	2(4.8)	0(0.0)	2(6.9)	0.852
No	40(95.2)	13(100.0)	27(93.1)	
Genitourinary				
Yes	20(47.6)	8(61.5)	12(41.4)	0.320
No	22(52.4)	5(38.5)	17(58.6)	
Single umbilical artery				
Yes	9(21.4)	2(15.4)	7(24.1)	0.816
No	33(78.6)	11(84.6)	22(75.9)	
Renal morphology changes ^**^				
Yes	12(28.6)	6(46.2)	6(20.7)	0.187
No	30(71.4)	7(53.8)	23(79.3)	
Pyelocalyceal dilatation				
Yes	7(16.7)	5(38.5)	2(6.9)	**0.037**
No	35(83.3)	8(61.5)	27(93.1)	
Central Nervous System				
Yes	27(64.3)	11(84.6)	16(55.2)	0.136
No	15(35.7)	2(15.4)	13(44.8)	
Ventriculomegaly				
Yes	13(31.0)	5(38.5)	8(27.6)	0.481
No	29(69.0)	8(61.5)	21(72.4)	
Holoprosencephaly				
Yes	9(21.4)	8(61.5)	1(3.4)	≤ 0.0001
No	33(78.6)	5(38.5)	28(96.6)	
Myelomeningocele				
Yes	9(21.4)	2(15.4)	7(24.1)	0.816
No	33(78.6)	11(84.6)	22(75.9)	
Agenesis of corpus callosum				
Yes	5(11.9)	3(23.1)	2(6.9)	0.326
No	37(88.1)	10(76.9)	27(93.1)	
Limbs abnormalities				
Yes	25(59.5)	6(46.2)	19(65.5)	0.400
No	17(40.5)	7(53.8)	10(34.5)	
Short bones				
Yes	6(14.3)	0(0.0)	6(20.7)	0.195
No	36(85.7)	13(100.0)	23(79.3)	
Clubfoot				
Yes	13(31.0)	3(23.1)	10(34.5)	0.705
No	29(69.0)	10(76.9)	19(65.5)	
Hands abnormalities				
Yes	20(47.6)	5(38.5)	15(51.7)	0.644
No	22(52.4)	8(61.5)	14(48.3)	
Diaphragmatic hernia				
Yes	7(16.7)	2(15.4)	5(17.2)	1.000
No	35(83.3)	11(84.6)	24(82.8)	
Cleft / palate lip				
Yes	10(23.8)	7(53.8)	3(10.3)	0.008
No	32(76.2)	6(46.2)	26(89.7)	

Legend: n: absolute frequency; n%: relative frequency; *p*: statistical significance; ^**^renal morphology changes involve cases of polycystic kidneys, renal dysplasias, and non specific renal changes; T13: trisomy 13; T18: trisomy 18; IAC: Inter Auricular Communication; IVC: Interventricular Communication; AVSD: Atrial Ventricular Septum Defect. ^*^Chi-square test with adjusted residual analysis. Frequencies in bold: association between variables by Chi-square test with adjusted residual analysis. Statistical significance was set as *p* ≤ 0.05 for all analyses.

The most common malformations found in fetuses with T13 were: holoprosencephaly (n=8, 61.5%), cleft lip and/or palate (n=7, 53.8%), renal morphology changes (including cystic kidneys, dysplastic kidneys, enlarged, hyperechogenic, and other non-specific kidney alterations) present in 6 (46.2%) cases, and ventricular septal defect (n=5, 38.5%). The most common morphological changes in T18 were: ventricular septal defect (n=15, 51.7%), hand defects (n=15, 51.7%), and clubfoot (n=10, 34.5%).

There was an increased incidence of cleft lip and/or palate (χ^2^ test, *p*=0.008), pyelocalyceal dilatation (χ^2^ test, *p*=0.037) and holoprosencephaly (χ^2^ test, *p* ≤ 0.0001) on T13 cases, corroborating data reported in the literature. The finding of short bones was exclusive of T18, corresponding to 6 (14.3%) cases.

### Pregnancy outcome and survival

The outcome of pregnancies with T13 and T18 is shown in Table 4. Gestational age at delivery was categorized into four groups, based on clinical relevance. Miscarriage was considered when the end of gestation occurred before 22 weeks + 6 days, because this is the limit gestational age of viability established in the Hospital de Clínicas of Porto Alegre; extremely preterm, those gestations that ended between 23 weeks and 31 weeks + 6 days; preterm, those whose end of gestation occurred between 32 weeks and 36 weeks + 6 days and at term, those pregnancies that reached 37 weeks or more.

**Table 4:** Natural history / outcomes for trisomy 13 and trisomy18.

Variable– *n*(*n*%)	Total *N*=42	T13 *n*=13	T18 *n*=29	^*^ *p-*value
Miscarriage^**^				
Yes	3(7.1)	0(0.0)	3(10.3)	0.579
No	39(92.9)	13(100.0)	26(89.7)	
Intra-uterine death				
Yes	39(92.9)	6(46.2)	15(51.7)	1.000
No	3(7.1)	7(53.8)	14(48.3)	
Live birth				
Yes	14(33.3)	7(53.8)	11(37.9)	0.531
No	28(66.7)	6(46.2)	18(62.1)	

Legend: n: absolute frequency; n%: relative frequency; *p*: statistical significance; ^**^miscarriage: considering the outcome before 22 + 6 weeks, limit of viability at the Hospital de Clínicas de Porto Alegre; T13: trisomy 13; T18: trisomy 18. ^*^Chi-Square test with adjusted residual analysis. Statistical significance was set at *p* ≤ 0.05 for all analyses.

The miscarriage rate for T18 was 9% (3/29) while there were no cases in T13. The fetal death rate was 46.2% (4/13) for T13 and 51.7% (15/29) for T18. The rate of live births was 54% (7/13) for T13; there was one extremely preterm birth (before 32 weeks), 57% (4/7) preterm births between 32 and 36 weeks + 6 days and 2 cases (28%) were at term pregnancies. The median (95% CI) survival of these infants was one day (33.55–90.40). Live births: 71% (5/7) died within the first 24 hours. There were two cases that exceeded the first week of life, one with 14 days and the other with 180 days.

For T18 the rate of live births was 37.9% (11/29), and 17% (2/11) of the cases were born extremely preterm, 56% (6/11) were preterm between 32 and 36 weeks + 6 days and 27% (3/11) of the cases were at term pregnancies. The median (95%CI) survival of these neonates was 2 days (-1.89–13.17). Five cases (45%) died within the first 24 hours and 45% (5/11) died in the first week of life. One case (10%) exceeded the first month of life, with a survival of 39 days. No case for both trisomies reached the first year of life.

**Figure 3:**
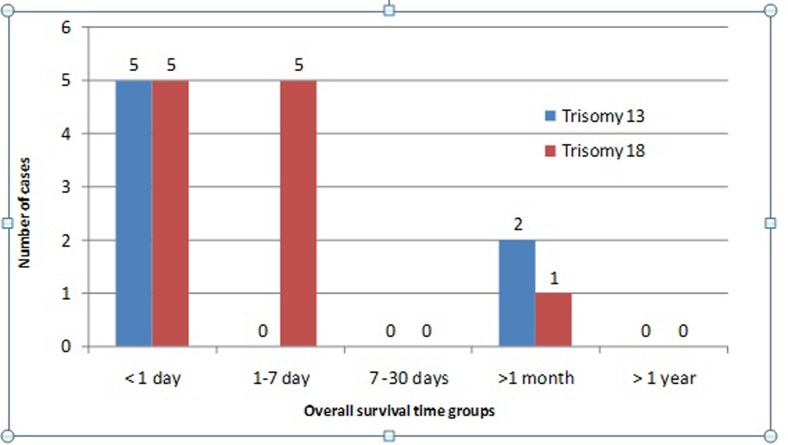
Offspring death distribution.

When analyzed on a case-by-case basis, it was observed that fetuses with greater survival were those in which neonatal investment was performed. They underwent multiple exams and procedures, including mechanical ventilation, surgical correction of some defects to guarantee vital functions, as feeding, breathing among others. Generally, their parents had a late diagnosis of trisomy, less time of prenatal care, and probably less time to prepare for mourning. Those couples with earlier diagnosis, with a better follow-up and counseling during the prenatal care, have opted for just a support or palliative management at birth for the newborn (Figure 3).

### Gestation at delivery and complications

Only 7.1% (3/42) of the total trisomies were miscarriages, and there were no cases within the T13 group, probably due to late diagnosis of pregnancy and a late prenatal referral.

Extremely preterm were 23.8% (10/42) of the cases, being 15.4% (2/13) for T13 and 27.6% (8/29) for T18. There were 54.8% (23/42) of premature cases, 69.2% (9/13) for T13 and 48.3% (14/29) for T18. Full-term gestation occurred in 14.3% (6/42) of the cases, with 15.4% (2/13) for T13 and 13.8% (4/29) for T18. When considering both trisomies, there was no difference between the gestational ages at the delivery (χ^2^ test, *p*=0.450).

The delivery route was analyzed for both groups, observing that 69% (29/42) of the births were vaginal delivered, occurring in 69.2% (9/13) of the T13 and in 69% (20/29) of the T18. Cesarean section (CS) was performed in 31% (13/42) of the trisomy cases, with 30.8% (4/13) for T13 and 31% (9/29) for T18. Some of the indications for CS were obstetric causes (e.g., abnormal fetal presentation, cesarean iterativity, and maternal contraindication for vaginal delivery). Despite prenatal counseling about fetal diagnosis, in few cases of non-reassuring fetal condition and parents desire to invest in the newborn, CS was also performed. There was no difference in the delivery route for the two trisomies (χ^2^ test, *p*=1.000).

Considering complications associated with pregnancy and birth, 21.4% (9/42) of the participants presented some intercurrence. Gestational hypertension disorders (e.g., gestational hypertension, pre-eclampsia, and pre-eclampsia superimposed on chronic hypertension) were the most observed, present in 11.9% (5/42) of the cases of trisomy. Prevalence of hypertensive disorders was 30.8% (4/13) in T13 cases and 3.4% (1/29) in T18, but with a marginal statistical difference (χ^2^ test, *p*=0.054).

Furthermore, when evaluating the length of maternal hospitalization stay after the final outcome of a trisomic gestation, 14.3% (5/42) had a maternal inpatient time of more than 3 days, and most of these cases occurred in mothers whose outcome of gestation was live birth. However, there was no statistical difference between the two trisomies (χ^2^ test, *p*=0.892).

## Discussion

This study describes the cases of T13 and T18 diagnosed in the past 23 years, at the Hospital de Clínicas in Porto Alegre, Porto Alegre/Brazil. Our cohort is one of the largest described so far in the literature that followed the natural history of these trisomies after prenatal diagnosis. All 42 of the included participants had prenatal diagnosis of full trisomy performed by amniocentesis for fetal karyotype.

Brazil is a country with a low resources health system (Monteklo and Aquino, 2011). This scenario is reflected in the quality of the pre-conception assessment and follow-up of pregnant women, mainly characterized by delay in the diagnosis of pregnancy, the late start of prenatal control, and inadequate availability of obstetric ultrasound equipment. An example of this is the absence of a universal and structured prenatal screening program for risk assessment of fetal aneuploidy. Our results show that pregnant women started the prenatal control mainly after 16 weeks of gestational age, making the first trimester screening for Down Syndrome and other aneuploidies not feasible.

Up to 70% of aneuploidy cases can be detected with combined ultrasound morphological markers (e.g., nuchal translucency and maternal risk factors) (Holmgren and Lacoursiere, 2008; Nicolaides, 2003), even in the absence of more complex methods that require greater resources, such as the use of biochemical markers, or complex ultrasound evaluations. Actions in order to increase availability of screening earlier in pregnancy have to be taken.

The main reason for the referral to the hospital was the presence of malformations detected by ultrasound, corresponding to 92.9% of the cases. The number and certain specific fetal malformations are directly associated with an increased risk-profile for aneuploidies; so obstetric sonographers need to know the pattern of malformations associated with each chromosomal anomaly (Nicolaides *et al.*, 1992). In our cases, it was possible to correlate some malformations and findings to each specific trisomy, thus increasing the diagnostic suspicion when detected in the ultrasound examination (Kroes *et al.*, 2014). This made it possible to correlate cleft lip and/or palate, holoprosencephaly and pyelocalyceal dilations with T13, which is consistent with data described in the literature (Patau *et al.*, 1960).

Among the 13 cases of T13 (47,+13) and 29 cases of T18 (47,+18) identified between October 1994 and October 2017, there was a difference regarding maternal age. In the T18 group most of women were over 35 years, similar to what is described in the literature (Pandya *et al.*, 1995; and Chitayat *et al.*, 2011), and there was no statically significant between the two trisomies in our study. When parity was considered, also no difference was found between primiparous and multiparous mothers.

When analyzing the natural history and the outcomes of these pregnancies, we observed approximately 50% of intrauterine fetal deaths for T13 and T18, and a short overall survival at birth for both conditions (above 50% death in the first 24 hours for live births). Thus, we confirm the potentially lethal condition of these polymalformative syndromes (Irving *et al.*, 2011, Lakovschek *et al.*, 2011; Houlihan and O’Donoghue*,* 2013).

One important finding is the high rate of cesarean section performed for T13 and T18 fetuses, including among primiparous women. This means that the counseling process offered to the parents should continue to be improved. The increased risk of emergency cesarean section compared to other types of delivery is well documented in the literature, as well as the potential implications of having an emergency cesarean section in future pregnancies (Villar *et al.*, 2006).

We observed that women who started late the prenatal control and with a late diagnosis of the aneuploidy had a higher incidence of cesarean delivery and more frequently chose to invest in the newborn.This cannot be affirmed without comparing C-section prevalence in Brazil and/or in similar settings, but this could be explained because these parents had less time for multidisciplinary team counseling, more difficulties to understand the fetal condition, and less time to elaborate mourning. The aim of counseling is always to provide complete information to the parents helping them in the decision-making process and respecting their autonomy. When the parents’ desire is to invest in the polymalformed newborn, the medical team offers an appropriate care based on the natural history of each condition. In this way, women who had a late diagnosis more frequently decided not to perform fetal necropsy and considered this pregnancy as a limitation to re-conceive.

Considering the newborns, greater survival was related to the number of procedures and interventions performed, but these did not enable them to reach the first year of life.

An important point for newborns with diagnosis of T13 or T18 is the management and care offered to them. Parents and health professionals must decide between support or palliative care without invasive procedures, or total investment. This is especially important in countries where the termination of pregnancy is not legal, or when parents decide to continue with the pregnancy. This decision has an impact not only for the newborn and the mother, but also reflects in costs for the health system. In a study done in the United Kingdom, it was estimated that children with malformations who underwent multiple surgeries, examinations, and procedures to survive 180 days could represent a daily hospitalization cost of approximately 1000 US dollars (Shetty *et al.*, 2016), depending on the type of procedure performed. However, despite such procedures, the natural history of the disease was not modified, because the interventions are not curative and only improve the quality of life.

The international guidelines for resuscitation and management of neonates with malformations (Wyckoff *et al.*, 2015) recommend providing specific palliative or supportive care, and always stressing that decisions must be taken together with the medical team, respecting the autonomy of the parents (Lin *et al.*, 2006; Nelson *et al.*, 2012; Shetty *et al.*, 2016). A positive impact in terms of public health and economic cost will be greater if some strategies were developed, such as the creation of structured evidence based-protocols considering the palliative and support strategies for newborns with aneuploidies.

Our results could support the reassessment of collective health policies and initiate the discussion about pregnancy termination for lethal polymalformative syndromes in a country where this kind of action is not legally approved. This is important from a public health perspective, as the termination of pregnancy could result in a positive impact on maternal morbidity/mortality and, secondarily, on the costs for the health system (Parmar *et al.*, 2015).

During the years in which the study was conducted, two types of decisions were taken. Some parents expressed their desire of pregnancy termination after diagnosis for T13 or T18. This request was made with judicial authorization for those cases of trisomy with severe and lethal malformations. On the other hand, many couples chose the option to continue with the pregnancy after diagnosis of an aneuploidy (Sibiude *et al.*, 2011; So *et al.*, 2017). Therefore, we emphasize the importance of structured protocols and trained multidisciplinary teams who perform an early diagnosis, an appropriate counseling process, and a quality follow-up to support the decision-making process.

This information regarding the natural history of pregnancies with prenatal diagnosis of T13 and T18 has local and international relevance, since it is associated with previous related studies and could improve the quantity and quality of information available for the counseling and follow-up processes. Local and international protocols or guidelines could be made to orientate the management of these pregnancies during the gestation period, delivery, and postpartum, considering both mother and newborn.

From the prenatal diagnosis of a fetus with T13 or T18, adequate clinical follow-up can be given to the parents and they can prepare for the probable outcome. An unexpected postnatal diagnosis can be extremely traumatic, as the parents may have little time to adjust to the reality of a child with significant malformations and the high risk of neonatal death. On the other hand, the parents could be assisted to take an informed decision-making process and thus determine together with a multidisciplinary team whether to maintain the pregnancy or requests its termination.

One limitation of our study is that the information contained in medical records is often incomplete or confusing. In addition, a recall bias could be present when it was necessary to establish telephone contact with the participants in order to get the information.

The main strength of the present study is associated with the diagnosis and inclusion criteria of the participants. All of the cases had prenatal diagnosis with full trisomy after amniocentesis for fetal karyotype. Cell culture was carried out by a biologist with experience in cytogenetics, and the follow-up and counseling were provided by the same multidisciplinary team formed by specialists in fetal medicine, experienced sonographers, clinical geneticists, psychologists, neonatologists, pediatricians, and complemented by the opinion of experts in pediatric surgery and pediatric urology. This allowed a homogeneous follow-up and management for these cases during those 20 years in which the study was performed. Notably, considering the worldwide prevalence and previous studies for T13 and T18 pregnancies, we have one of the largest series of cases.

## Conclusions

The results of this study confirm the bad prognosis for fetuses with trisomy 13 or 18. More than 50% of intrauterine death occurred and, among live births, a short postnatal life was observed with a median survival time of one day for T13 and two days for T18. For both trisomies, no patient reached the first year of life. For those who had a longer survival, multiple invasive and expensive procedures were required without success in prolonging life beyond 180 days.

The results of this study are consistent with those referenced in the literature, emphasizing the need of structured protocols and guidelines aiming at early T13 and T18 diagnosis, prenatal care, gestation/parents follow-up, and counseling processes. For those couples with earlier diagnosis, a better follow-up and counseling during the prenatal care lead to the option for a support or palliative management of the newborn.

Current and local data about the natural history of T13 and T18 are necessary. Also, this data can contribute to include new therapeutic options in the actual legislation. Finally, when the counseling process is appropriate, it becomes easier to take decisions respecting the parent’s autonomy and to look for better outcomes for both, the mother and the fetus.
